# Real-world application of disitamab vedotin (RC48-ADC) in patients with breast cancer with different HER2 expression levels: efficacy and safety analysis

**DOI:** 10.1093/oncolo/oyae304

**Published:** 2024-11-16

**Authors:** Ke Wang, Ting Xu, Jing Wu, Yuan Yuan, Xiaoxiang Guan, Chengjun Zhu

**Affiliations:** Department of Oncology, The First Affiliated Hospital of Nanjing Medical University, Nanjing 210009, People’s Republic of China; Department of Chemotherapy, Jiangsu Cancer Hospital, Jiangsu Institute of Cancer Research, The Affiliated Cancer Hospital of Nanjing Medical University, Nanjing 210009, People’s Republic of China; Department of Oncology, The First Affiliated Hospital of Nanjing Medical University, Nanjing 210009, People’s Republic of China; Department of Chemotherapy, Jiangsu Cancer Hospital, Jiangsu Institute of Cancer Research, The Affiliated Cancer Hospital of Nanjing Medical University, Nanjing 210009, People’s Republic of China; Department of Oncology, The First Affiliated Hospital of Nanjing Medical University, Nanjing 210009, People’s Republic of China; Jiangsu Key Lab of Cancer Biomarkers, Prevention and Treatment, Collaborative Innovation Center for Personalized Cancer Medicine, Nanjing Medical University, Nanjing 210029, People’s Republic of China; Department of Oncology, The First Affiliated Hospital of Nanjing Medical University, Nanjing 210009, People’s Republic of China

**Keywords:** breast cancer, HER2, antibody-drug conjugate, disitamab vedotin

## Abstract

**Background:**

Disitamab vedotin (RC48-ADC), an antibody-drug conjugate (ADC), combines specific antibody disitamab with cytotoxicity monomethyl auristatin E to effectively target the human epidermal growth factor receptor 2 (HER2) protein on tumor cells for precise elimination. Recent studies have demonstrated that RC48-ADC offers therapeutic benefits for patients with HER2-positive and HER2-low-expression breast cancer (BC). However, a thorough exploration of its efficacy and safety in real-world settings for patients with metastatic breast cancer (mBC) is currently lacking.

**Methods:**

This retrospective, multicenter, real-world study included patients with mBC who received RC48-ADC from September 2021 to March 2024. These patients include HER2-positive BC and HER2-low-expression BC. The primary endpoint was progression-free survival (PFS). Secondary endpoints included overall survival (OS), restricted mean survival time, objective response rate (ORR), and disease control rate (DCR). Factors affecting efficacy and the occurrence of treatment-related adverse events (TRAE) were evaluated.

**Results:**

The study included a cohort of 89 patients with mBC, with 48 of those being identified as HER2-positive. As of March 2024, 22 deaths were recorded, with an immature median OS. Total PFS varied from 1.0 to 31.2 months, with a median of 5.5 months (95% CI, 4.368-6.632). HER2-positive patients exhibited prolonged PFS compared with HER2-low-expression patients (6.6 months vs 4.1 months, *P *= .023). The overall ORR stood at 25.8% (95% CI, 0.178-0.358), with higher rates observed in HER2-positive patients compared with HER2-low-expression patients (31.3% vs 19.5%). Similarly, the overall DCR was 78.7% (95% CI, 0.691-0.859), with HER2-positive patients demonstrating superior DCR compared with HER2-low-expression patients (83.3% vs 73.2%). Notably, HER2 expression emerged as the primary determinant of RC48-ADC efficacy. The most prevalent TRAE among all patients included leukopenia (21.3%) and alopecia (20.2%).

**Conclusion:**

RC48-ADC showcases promising efficacy and manageable safety in patients with both HER2-positive and HER2-low-expression mBC.

**Implications for Practice**

Our retrospective study on patients receiving the novel antibody-drug conjugate (ADC), disitamab vedotin (RC48-ADC), confirms positive therapeutic response and manageable safety in human epidermal growth factor receptor 2 (HER2)-positive and low-expression patients. Moreover, as a follow-up treatment option post other ADC therapies, RC48-ADC exhibits encouraging efficacy.

## Introduction

Breast cancer (BC) stands out as a prevalent malignancy affecting women’s health globally.^1^ According to GLOBOCAN 2020 statistics, the worldwide age-standardized incidence and mortality rates (per 100 000 women) can reach up to 47.8 and 13.6, respectively.^2^ Among various subtypes, human epidermal growth factor receptor 2 (HER2)-positive BC accounts for approximately 15%-20% of cases and exhibits highly aggressive biological features.^3^ HER2 is a proto-oncogene that encodes a transmembrane receptor tyrosine kinase, belonging to the ErbB receptor family. HER2 plays a pivotal role in the pathogenesis of BC and other malignancies, with its expression or gene amplification frequently correlated with aggressive disease phenotypes and poor clinical outcomes.^4^ Consequently, HER2-positive is used to describe tumor cells exhibiting HER2 protein expression or HER2 gene amplification, defined as immunohistochemistry (IHC)3+ or IHC2+ with fluorescence in situ hybridization (FISH) positivity.^5^ Importantly, the elevated expression of HER2 on the surface of tumor cells presents a therapeutic target for the development of specific anti-HER2 agents. Patients with HER2-positive BC generally demonstrate a robust therapeutic response to HER2-targeted therapies,^6,7^ encompassing monoclonal antibodies (eg, trastuzumab, pertuzumab), small-molecule tyrosine kinase inhibitors (TKIs) (eg, lapatinib, neratinib), and antibody-drug conjugates (ADCs) (eg, Trastuzumab deruxtecan, Trastuzumab emtansine).^6,8^ Conversely, HER2-low-expression refers to a level of HER2 protein expression that is intermediate between positive and totally negative, typically characterized by an IHC score of 1+ or 2+ with FISH negative. Although HER2-low-expression tumors have historically been excluded from traditional HER2-targeted therapies, this subgroup is increasingly recognized as a distinct entity, warranting further investigation into their potential responsiveness to novel anti-HER2 therapeutic strategies.

ADCs represent a groundbreaking category of anticancer therapies, combining the targeting specificity of monoclonal antibodies with the cell-killing efficacy of cytotoxic drugs.^9^ This synergy aims to precisely identify and selectively eliminate tumor cells while minimizing adverse effects on normal tissues, thereby enhancing therapeutic efficacy and reducing side effects.^10^ The conceptual framework of ADCs relies on the capacity of monoclonal antibodies to selectively recognize and bind to antigens expressed on the surface of cancer cells.^11,12^ Upon binding to the target antigen, ADCs undergo internalization into tumor cells and exert cytotoxic effects via different mechanisms, ultimately precipitating tumor cell demise.^13-15^ The type of linker in ADCs significantly affects their therapeutic mechanism. Cleavable linkers, as seen with Trastuzumab deruxtecan (T-DXd), result in the release of cytotoxic payloads that can penetrate cell membranes and induce the death of adjacent tumor cells, known as the bystander effect. In contrast, non-cleavable linkers, used in Trastuzumab emtansine (T-DM1), do not exhibit a bystander effect.^12^ These differences affect the choice of ADCs and their treatment outcomes.

HER2-directed ADCs represent a significant class of targeted therapies designed to deliver cytotoxic agents specifically to HER2-expressing tumor cells. These ADCs differ in their cytotoxic payloads, linkers, and mechanisms of action. For instance, T-DM1 combines the HER2-targeting monoclonal antibody trastuzumab with the cytotoxic agent emtansine (DM1) via a stable thioether linker, demonstrating effectiveness in HER2-positive BC and improved survival outcomes in clinical trials.^16^ The advent of anti-HER2 agents has significantly improved the prognosis of individuals with HER2-positive BC. Nonetheless, patients with HER2-low-expression BC, comprising 45%-55% of BC cases, frequently derive limited benefit from conventional anti-HER2 medications.^17,18^ Recent clinical studies on novel anti-HER2 ADCs have shown promising therapeutic efficacy in patients with HER2-low-expression BC, signaling a shift in their treatment landscape.^19-21^ DESTINY-Breast04 (DB-04) is the first phase III clinical trial to achieve positive results for patients with HER2-low-expression metastatic BC. This study showed that T-DXd significantly improves progression-free survival (PFS) and overall survival (OS) compared with the physician’s choice of chemotherapy, regardless of hormone receptor status, with manageable safety. T-Dxd combines trastuzumab with the topoisomerase I inhibitor deruxtecan, and its efficacy in HER2-low BC has led to its approval for use in such cases, providing a new therapeutic option for patients who previously had limited treatment choices.^22^ However, after a period of treatment, the patient may still develop drug resistance, necessitating a change to a new treatment regimen.

Disitamab vedotin (RC48-ADC), the pioneering HER2-targeted ADC in China, comprises a novel humanized anti-HER2 monoclonal antibody covalently linked to the microtubule inhibitor monomethyl auristatin E (MMAE) via a cleavable linker.^23^ It has obtained regulatory approval for the treatment of HER2 IHC2+ or IHC3+ gastric cancer and urothelial cancer in China.^24-26^ The indication for BC is also under review for approval. A study conducted by Wang Jiayu et al on phase I/Ib clinical trials of RC48 in BC demonstrated substantial efficacy in patients with both HER2-positive and HER2-low-expression advanced BC. At a dose of 2.0 mg/kg administered every 2 weeks, the confirmed objective response rates were 42.9% for patients with HER2-positive BC and 33.3% for patients with HER2-low-expression BC, with median PFS durations of 5.7 and 5.1 months, respectively. Additionally, the study established a promising safety profile, with common grade 3 or higher adverse events including neutropenia and fatigue.^27^ Recent research suggests that RC48 exhibits favorable efficacy in patients with both HER2-positive and HER2-low-expression BC, without any new safety concerns.^28^

Therefore, to further explore the efficacy and safety of RC48 in real-world clinical practice, this study retrospectively analyzed the clinical records of 89 patients with metastatic BC, either HER2-positive or HER2-low-expression, treated with RC48 at Nanjing Medical University First Affiliated Hospital and Jiangsu Cancer Hospital. By closely observing treatment response and the incidence of adverse events throughout the therapeutic course, the aim of this investigation is to provide a fundamental reference for subsequent clinical discussions and decision-making processes.

## Materials and methods

### Study design and patients

**Figure 1.**
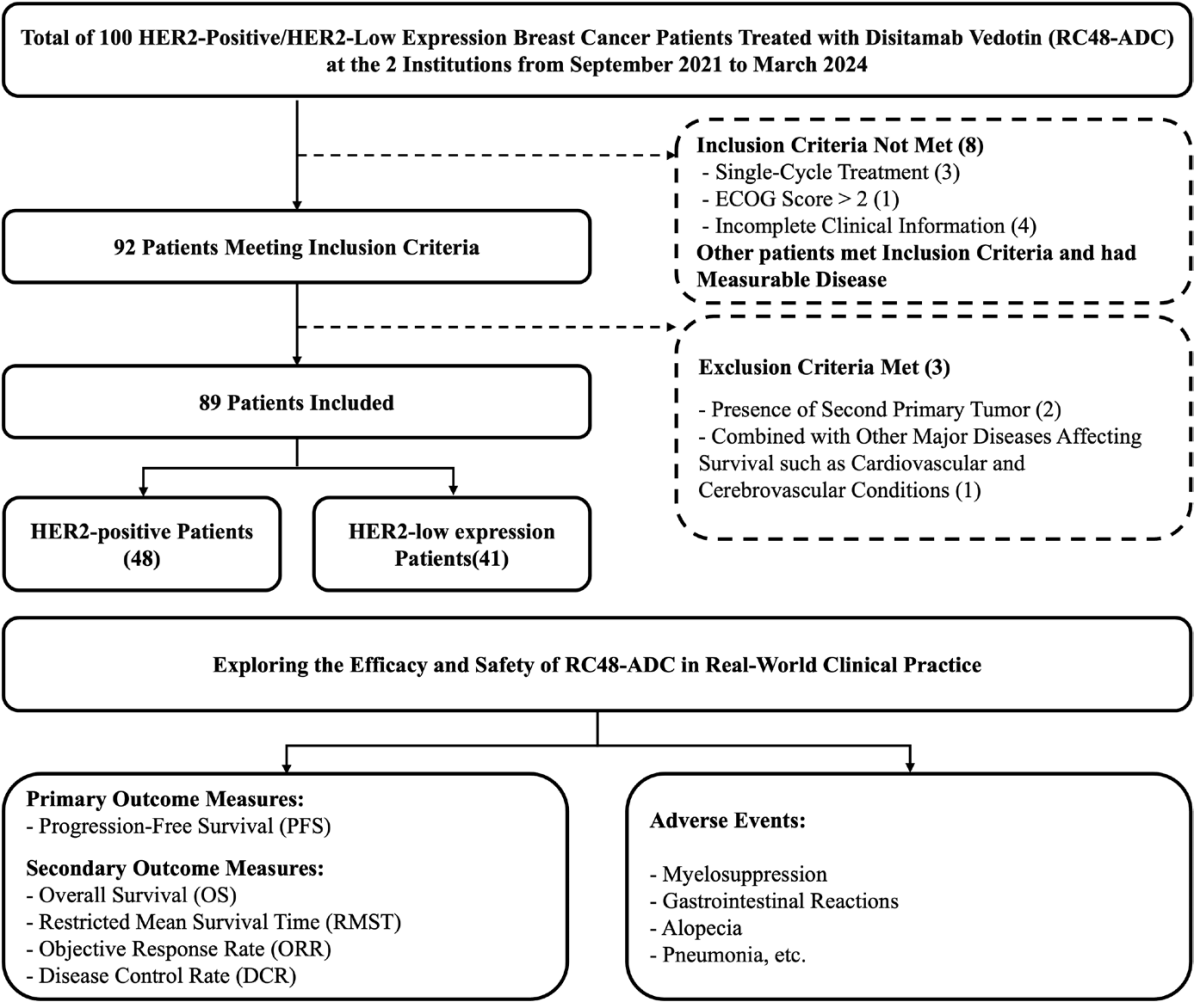
Flowchart of research design.

A review of the medical records from Jiangsu Provincial People’s Hospital and Jiangsu Cancer Hospital was conducted to identify patients diagnosed with HER2-positive or HER2-low-expression BC who received treatment with RC48 (Figure 1). Eligible patients were aged 18-80 years and were required to have a histologically confirmed diagnosis of HER2-positive or HER2-low-expression BC. Patients who received at least 2 cycles of RC48 rescue therapy between September 2021 and March 2024 were included in the analysis. Additionally, patients were required to have an Eastern Cooperative Oncology Group (ECOG) performance status of ≤2 and complete clinical data available. According to the Response Evaluation Criteria in Solid Tumors (RECIST, version 1.1), patients must have at least one measurable extracranial lesion or evidence of lytic or mixed bone metastases. Patients with a secondary primary tumor, male BC, bilateral BC, major concomitant diseases affecting survival (such as cardiovascular or cerebrovascular diseases), hepatic or renal failure, pregnancy or lactation, or other conditions deemed unsuitable by the investigators were excluded from the study. The institutional Review Board of the First Affiliated Hospital with Nanjing Medical University has given approval to the protocol (2024-SR-264). In addition, this study followed the Declaration of Helsinki.

### Data collection and definitions

To evaluate the efficacy of RC48 in a real-world population of patients with HER2-positive and HER2-low-expression BC, we conducted a retrospective chart review of electronic medical records. These patients received a standard dose of 2.0 mg/kg via intravenous infusion every 2 weeks, except some were extended to every 3 weeks because of adverse effects.

Baseline characteristics included age, height, weight, body mass index (BMI), menstrual status, TNM staging, and ECOG performance status. Clinical characteristics included initial staging, surgical status, histopathological type, HER2 expression status, molecular subtype, metastatic status (specific metastatic sites, whether singular or multiple), previous treatment regimens, imaging examination results, and RC48 treatment regimen. At the time of initial diagnosis and upon the emergence of new lesions, HER2 expression of the tumor was determined through pathological IHC and FISH testing and based on the most recent pathological results. BC molecular subtypes are primarily classified based on the expression of estrogen receptor, progesterone receptor, and HER2. By assessing the positivity or negativity of these markers, BC can be categorized into luminal, HER2-positive (HER2+), and triple-negative BC (TNBC). Additionally, all TNM staging mentioned in the article refers to the stage at the time of initial diagnosis.

Patients were assessed every 2 cycles using a combination of clinical evaluations and imaging scans. In addition, tumor evaluation was evaluated every 6 weeks when their condition was stable to minimize radiation damage. The assessment methods typically included computed tomography (CT) scans, magnetic resonance imaging (MRI), and/or positron emission tomography (PET) scans. Efficacy assessments were conducted by the clinical physicians according to the RECIST 1.1.^29^ Response categories included: complete response (CR), partial response (PR), stable disease (SD), and progressive disease (PD). The primary endpoint of efficacy assessment in this study was progression-free survival (PFS). The second endpoint included overall survival (OS), restricted mean survival time (RMST), objective response rate (ORR), and disease control rate (DCR). Definitions were as follows: PFS: time from initiation of treatment to first disease progression or death from any cause, whichever comes first; OS: time from initiation of treatment to death or last follow-up; RMST: The mean survival time of the study cohort within a specified follow-up duration; ORR: percentage of patients achieving CR or PR; DCR: percentage of patients achieving CR, PR, or SD according to RECIST 1.1. Treatment-related adverse events (TRAE) were graded according to the National Cancer Institute Common Terminology Criteria for Adverse Events (NCI-CTCAE 5.0).^30^

### Statistical analysis

Data were analyzed using SPSS 26.0, GraphPad Prism 9.0, and R software 4.0.1. Categorical data are expressed as percentages and between-group differences were compared using the χ^2^ test. Kaplan-Meier method was used to plot survival curves with a log-rank test for comparison. The significance level was set at *α* = 0.05, and all *P*-values and confidence intervals were based on 2-tailed tests, with *P* < .05 considered statistically significant.

## Result

### All patients

As of March 2024, a total of 89 patients with HER2-positive or HER2-low-expression BC were included in our study (Table 1). Their median age was 53 years (ranging from 28 to 80 years) with a median BMI of 22.89 (ranging from 14.92 to 30.48). The follow-up period ranged from 2.3 to 31.2 months, with a median follow-up time of 16.5 months. The molecular subtypes included 29 cases (32.6%) of luminal, 48 cases (53.9%) of HER2-positive, and 12 cases (13.5%) of TNBC. Among the patients, 48 (53.9%) had HER2-positive tumors, and 41 (46.1%) had HER2-low tumors. There was a total of 32 patients (36.0%) with brain metastases, among whom 12 had undergone radiation therapy or surgery, while 20 had not received the above treatment. RC48 was predominantly used as third-line or later therapy for patients with HER2-positive or HER2-low-expression BC, accounting for 78.7% (70/89). The median total dose of RC48 per cycle in all patients was 120 mg, with the number of treatment cycles ranging from 2 to 32 cycles and a median of 5 cycles.

**Table 1.** Characteristics of all patients.

Characteristics	Total	HER2-positive	HER2-low expression
Total [*n* (%)]	89 (100)	48 (100)	41 (100)
Age [median (range), years]	53 (28-80)	55 (28-69)	53 (31-80)
BMI [median (range), kg/m^2^]	22.89 (14.92-30.48)	22.86 (15.06-30.48)	22.89 (14.92-29.43)
Menstrual status at initial diagnosis [*n* (%)]			
Pre-menopausal	29 (32.6)	12 (25.0)	17 (41.5)
Post-menopausal	60 (67.4)	36 (75.0)	24 (58.5)
Initial diagnosis stage [*n* (%)]			
Unknown	5 (5.6)	4 (8.3)	1 (2.4)
Stage I	8 (9.0)	5 (10.4)	3 (7.3)
Stage II	29 (32.6)	13 (27.1)	16 (39.0)
Stage III	34 (38.2)	18 (37.5)	16 (39.0)
Stage IV	13 (14.6)	8 (16.7)	5 (12.2)
Hormone receptor status [*n* (%)]			
Negative	36 (40.4)	24 (50.0)	12 (29.3)
Positive	53 (59.6)	24 (50.0)	29 (70.7)
Molecular subtypes [*n* (%)]			
Luminal	29 (32.6)	0 (0)	29 (70.7)
HER2-positive	48 (53.9)	48 (100)	0 (0)
TNBC	12 (13.5)	0 (0)	12 (29.3)
Total number of distant metastases [*n* (%)]			
1	27 (30.3)	15 (31.3)	12 (29.3)
More than 1	62 (69.7)	33 (68.7)	29 (70.7)
Locations of distant metastases [*n* (%)]			
Brain	32 (36.0)	22 (45.8)	10 (24.4)
Liver	44 (49.4)	25 (52.1)	19 (46.3)
Bone	51 (57.3)	26 (54.2)	25 (61.0)
Lung	49 (55.1)	28 (58.3)	21 (51.2)
Others	38 (42.7)	17 (35.4)	21 (51.2)
Previous anti-HER2 therapy [*n* (%)]			
Yes	50 (56.2)	45 (93.8)	5 (12.2)
No	39 (43.8)	3 (6.3)	36 (87.8)
Previous other ADC drugs [*n* (%)]			
None	79 (88.8)	40 (83.3)	39 (95.1)
DS-8201	2 (2.2)	0 (0)	2 (4.9)
T-DM1	8 (9.0)	8 (16.7)	0 (0)
Previous anti-microtubule agents [*n* (%)]			
Yes	84 (94.4)	45 (93.8)	39 (95.1)
No	5 (5.6)	3 (6.3)	2 (4.9)
Lines of therapy [*n* (%)]			
First line	5 (5.6)	2 (4.2)	3 (7.3)
Second line	14 (15.7)	7 (14.6)	7 (17.1)
Third line and above	70 (78.7)	39 (81.2)	31 (75.6)
Combination of therapy [*n* (%)]			
Monotherapy	33 (37.1)	22 (45.8)	11 (26.8)
Chemotherapy	12 (13.5)	4 (8.3)	8 (19.5)
Targeted therapy	33 (37.0)	19 (39.6)	14 (34.1)
Other therapy	11 (12.4)	3 (6.3)	8 (19.5)
Treatment cycle [*n* (%)]			
14 days	68 (76.4)	38 (79.2)	30 (73.2)
21 days	21 (23.6)	10 (20.8)	11 (26.8)

**Figure 2.**
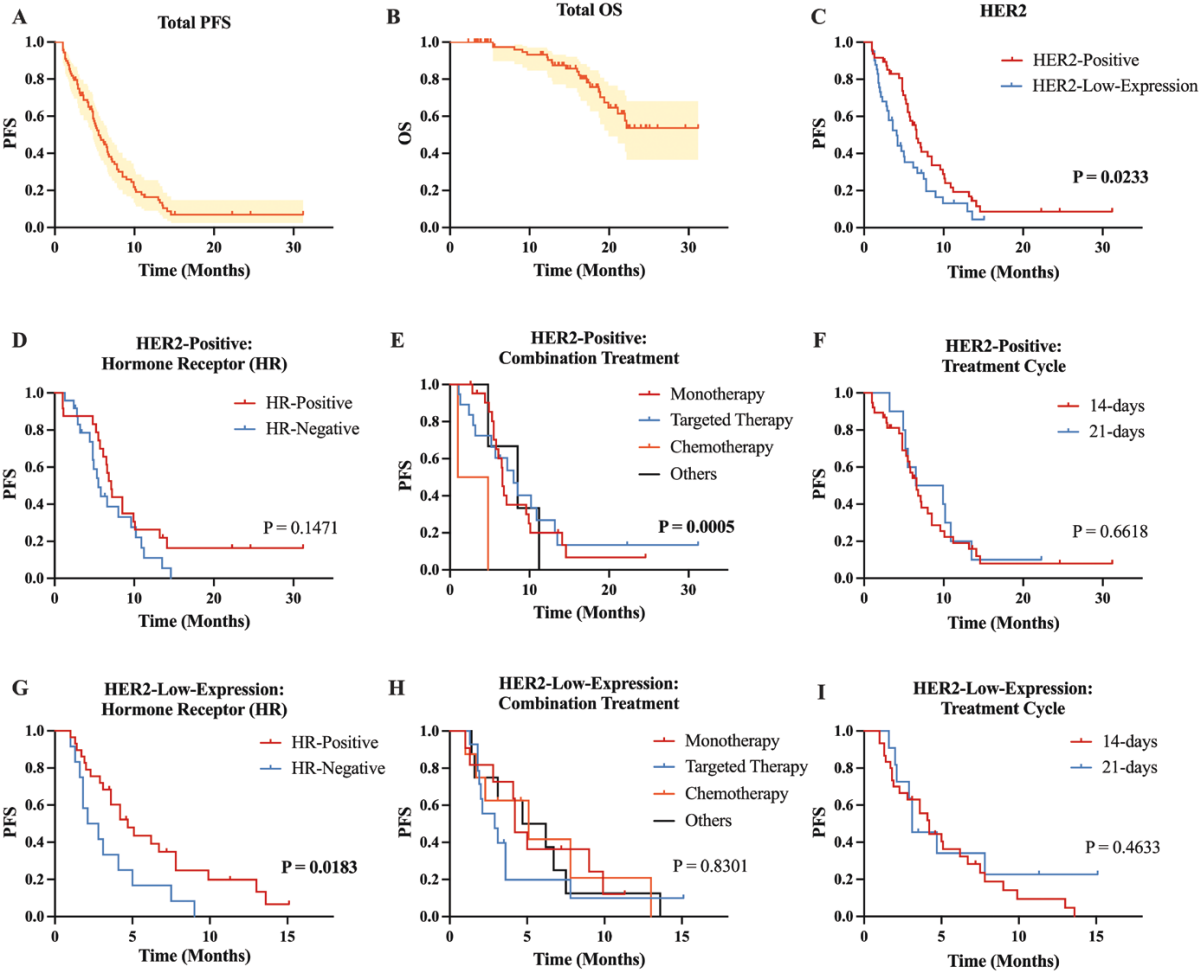
Survival plots of all patients and subgroups with different HER2 expression levels. Progression-free survival (PFS) (A) and overall survival (OS) (B) for all patients. Comparison of PFS in patients with HER2 positive and HER2-low-expression breast cancer (BC) (C). Comparison of PFS in patients with HER2-positive BC with different hormone receptor (HR) status (D), combination treatment types (E), and time of treatment cycle (F). Comparison of PFS in patients with HER2-low-expression BC with different hormone receptor (HR) status (G), combination treatment types (H), and time of treatment cycle (I).

The median PFS for the 89 patients was 5.5 months (95% CI, 4.368-6.632, Figure 2A). Among the best responses of the 89 patients, 4 cases (4.5%) achieved CR, 19 cases (21.3%) achieved PR, 47 cases (52.8%) achieved SD, and 19 cases (21.3%) experienced PD (Table 2). The ORR was 25.8% (23/89, 95% CI, 0.178-0.358), and the DCR was 78.7% (70/89, 95% CI, 0.691-0.859). Additionally, a total of 22 patients had reached the OS endpoint, but the median OS had not yet been reached (Figure 2B). Considering that patients with HER2-positive and HER2-low-expression BC represent 2 distinct populations, we compared their PFS, and the results showed a statistically significant difference (6.6 vs 4.1 months, *P* = .023, Figure 2C).

**Table 2.** Best response and outcomes of all patients.

Best response and outcomes	Total	HER2-positive	HER2-low expression
Total	89 (100)	48 (100)	41 (100)
Complete response (CR) [*n* (%)]	4 (4.5)	3 (6.3)	1 (2.4)
Partial response (PR) [*n* (%)]	19 (21.3)	12 (25.0)	7 (17.1)
Stable disease (SD) [*n* (%)]	47 (52.8)	25 (52.1)	22 (53.7)
Progressive disease (PD) [*n* (%)]	19 (21.3)	8 (16.7)	11 (26.8)
Overall response rate (ORR) (%)	25.8	31.3	19.5
Disease control rate (DCR) (%)	78.7	83.3	73.2

### Patients with HER2-positive breast cancer

In our retrospective study, a total of 48 patients with HER2-positive BC were included, with a median PFS of 6.6 months (95%CI, 5.365-7.835, Table 3). Among the best treatment responses of the 48 patients, 3 cases (6.3%) achieved CR, 12 cases (25.0%) achieved PR, 25 cases (52.1%) achieved SD, and 8 cases (16.7%) showed PD. The ORR for patients with HER2-positive BC was 31.3% (15/48, 95% CI, 0.200-0.454, Table 2) and the DCR was 83.3% (40/48, 95% CI, 0.704-0.913, Table 2).

**Table 3.** The influencing factor of PFS.

Clinical characters	HER2-positive		HER2-low expression	
	Median PFS (months)	*P*-value	Median PFS (months)	*P*-value
Total	6.6	—	4.1	—
Hormone receptor status (positive/negative)	7.1/5.5	0.147	4.7/2.1	**0.018** ^b^
Brain metastasis (yes/no)	5.1/5.0	0.957	4.1/3.6	0.815
Liver metastasis (yes/no)	6.5/8.0	0.435	4.2/3.6	0.605
Bone metastasis (yes/no)	7.1/6.5	0.306	4.7/3.1	0.473
Lung metastasis (yes/no)	6.6/7.1	0.298	4.2/3.6	0.999
Combination of therapy^a^	6.6/1.0/8.0/8.5	**<0.001** ^b^	4.2/5.1/2.9/4.7	0.830
Treatment cycle (14 days/21 days)	6.6/6.5	0.662	4.2/3.1	0.463

^a^Combination of therapy (monotherapy/chemotherapy/targeted therapy/others).

^b^The bold *P*-value indicates statistical significance.

The median age of the 48 patients was 55 years (range from 28 to 69), and the median BMI was 22.86 (range from 15.06 to 30.48). Among them, 24 patients (50%) were hormone receptor (HR)-positive, while 24 patients (50%) were HR-negative. The PFS for the 2 groups was 7.1 months and 5.5 months, respectively, with no statistically significant difference (*P* = 0.147, Figure 2D, Table 3). Prior to receiving RC48 treatment, 15 patients (31.3%) had one site of distant metastasis, while 33 patients (68.7%) had more than one metastatic site. The most common sites of metastasis were the lungs (28 patients, 58.3%), followed by the bones (26 patients, 54.2%), liver (25 patients, 52.1%), brain (22 patients, 45.8%), and other organs (17 patients, 35.4%), including the pleura, chest wall, abdominal wall, and adrenal glands. There was no significant difference in PFS based on the presence or absence of metastasis in these organs (Supplementary Figure S1A-D, Table 3).

Among patients with HER2-positive treated with RC48, 22 patients (45.8%) received monotherapy, while 26 patients (54.2%) received combination therapy, including 4 patients (8.3%) with chemotherapy, 19 patients (39.6%) with targeted therapy, and 3 patients (6.3%) with other combination therapies (including 2 patients receiving both chemotherapy and targeted therapy and 1 patient receiving endocrine therapy). The PFS for monotherapy was 6.6 months (95% CI, 6.165-7.035), while the PFS for patients receiving combination chemotherapy was 1.0 months (95% CI, 0.750-5.050), combination-targeted therapy was 8.0 months (95% CI, 4.626-11.374), and combination of other therapies was 8.5 months (95% CI, 2.579-14.421). These results suggest a statistically significant difference in PFS among monotherapy and combination therapies (*P* < .01, Figure 2E, Table 3), with combination-targeted therapy and other combination therapies showing longer PFS.

Reviewing medical records, 38 patients (79.2%) received the standard 14-day cycle, while 10 patients (20.8%) were treated with a 21-day cycle due to poor performance status and adverse reactions during initial treatment. There was no significant difference in PFS between the 2 groups (6.6 vs 6.5 months, *P* = 0.662, Figure 2F, Table 3).

### Patients with HER2-low-expression BC

Additionally, 41 patients with HER2-low-expression BC received RC48 treatment, with a median PFS of 4.1 months (95% CI, 2.850-5.350, Table 3). Among the best treatment responses of the 41 patients, 1 case (2.4%) achieved CR, 7 cases (17.1%) achieved PR, 22 cases (53.7%) achieved SD, and 11 cases (26.8%) experienced PD. The ORR for patients with HER2-low-expression BC was 19.5% (8/41, 95% CI, 0.102-0.340, Table 2), and the DCR was 73.2% (30/41, 95% CI, 0.581-0.843, Table 2).

The median age of the 41 patients was 53 years (range 31-80), and the median BMI was 22.89 (range 14.92-29.43). Among them, 29 patients (70.7%) were HR-positive, while 12 patients (29.3%) were HR-negative. The median PFS for patients with HR-positive BC was 4.7 months (95% CI, 2.977-6.423), and for patients with HR-negative BC, it was 2.1 months (95% CI, 0.403-3.797), showing a significant difference (*P* = 0.018, Figure 2G, Table 3). Prior to receiving RC48 treatment, 12 patients (29.3%) had one site of distant metastasis, while 29 patients (70.7%) had more than 1 metastatic site. The most common sites of metastasis were the bones (25 patients, 61.0%), followed by the lungs (21 patients, 51.2%), liver (19 patients, 46.3%), brain (10 patients, 24.4%), and other organs (21 patients, 51.2%), including the pleura, chest wall, abdominal wall, and adrenal glands. There was no significant difference in PFS based on the presence or absence of metastasis in these organs (Supplementary Figure S1E-H, Table 3).

Among patients with HER2-low-expression BC, 11 patients (26.8%) received monotherapy, while 30 patients (73.2%) received combination therapy, including 8 patients (19.5%) with chemotherapy, 14 patients (34.1%) with targeted therapy, and 8 patients (19.5%) with other combination therapies, including 5 patients receiving both chemotherapy and targeted therapy, 2 patients receiving endocrine therapy, and 1 patient receiving immunotherapy. The PFS for monotherapy was 4.2 months (95% CI, 3.229-5.171), the PFS for patients receiving combination chemotherapy was 5.1 months (95% CI, 0.000-10.496), the PFS for patients receiving combination-targeted therapy was 2.9 months (95% CI, 1.195-4.605), and the PFS for those receiving other combination therapies was 4.7 months (95% CI, 0.404-8.996). The results showed no significant difference between monotherapy and combination therapy, and unlike in the HER2-positive group, combination-targeted therapy did not demonstrate favorable outcomes (*P* = .83, Figure 2H, Table 3).

In patients with HER2 low expression, 30 patients (73.2%) were treated with the standard 14-day cycle, while 11 patients (26.8%) were treated with a 21-day cycle. There was no significant difference in PFS between the 2 groups (4.2 vs 3.1 months, *P* = .463, Figure 2I, Table 3).

### RMST and sequential treatment with ADCs

**Figure 3.**
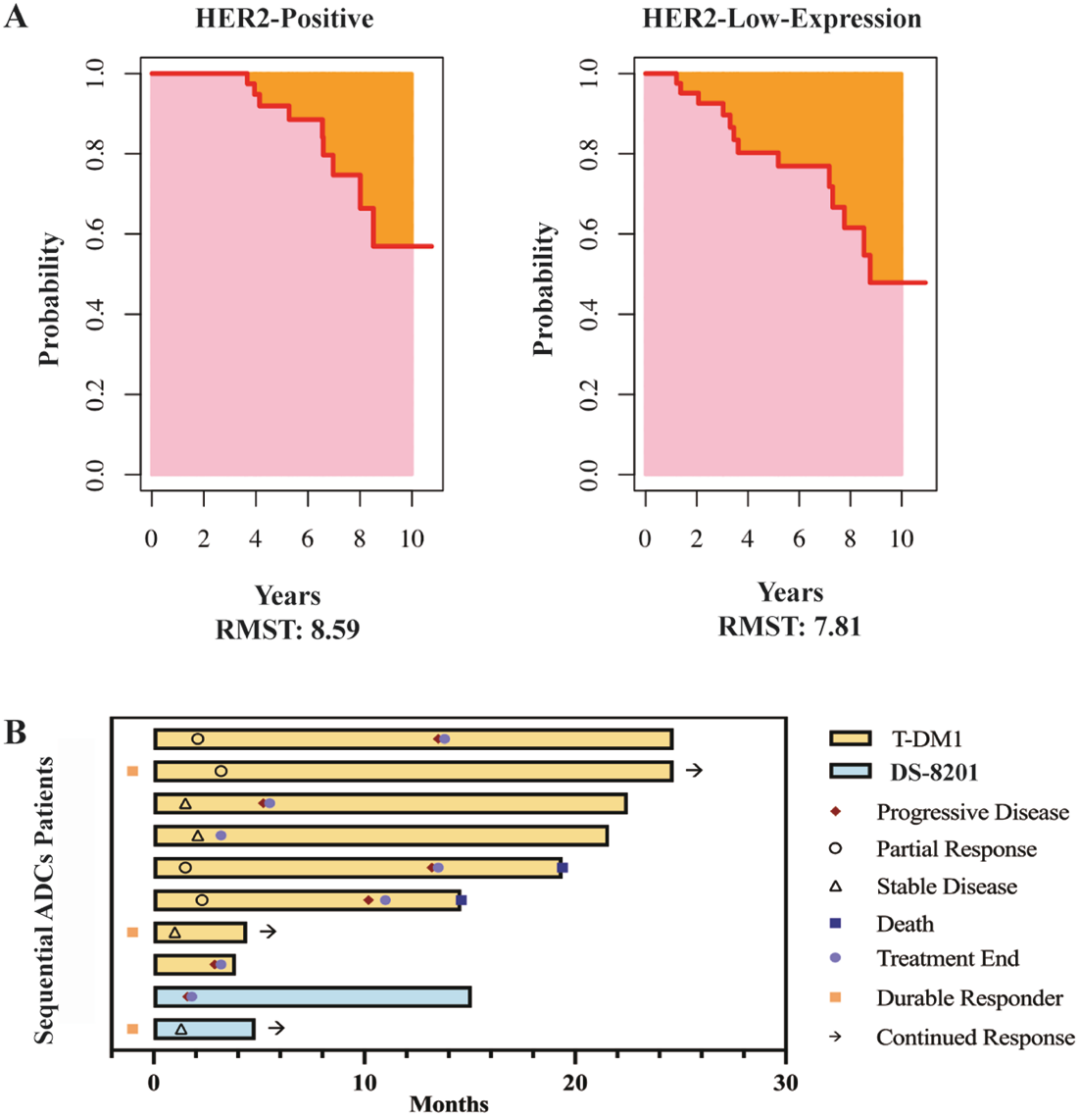
Survival outcomes and sequential therapy in patients with HER2-positive and HER2-low-expression BC. Comparison of restricted mean survival time between patients with HER2-positive and HER2-low-expression BC (A). Swimmer diagram of T-DM1 and DS-8201 followed by RC48 therapy (B).

Additionally, to compare patients with HER2-positive and HER2-low-expression BC, we calculated the 10-year RMST. The results showed that patients with HER2-positive BC had a longer RMST compared with patients with HER2-low-expression BC (8.6 vs 7.8 years), although the difference was not statistically significant (Figure 3A). Notably, among the patients with HER2-positive BC, 8 patients (16.7%) had previously received another ADC drug, T-DM1, followed by RC48 treatment. Two of these patients remained progression-free at their most recent follow-up, at 3.2 and 6.3 months, respectively, while the remaining 6 patients had PFS ranging from 2.9 to 13.5 months, with a median PFS of 10.2 months (95% CI, 0.238-20.162). Among patients with HER2-low-expression BC, 2 patients (4.8%) had previously received another ADC drug, DS-8201. One patient remained progression-free at the most recent follow-up (3.5 months), while the other patient had a PFS of 1.6 months (Figure 3B).

### Safety

Common TRAE among all patients included myelosuppression, alopecia, gastrointestinal reactions, and hepatic dysfunction (Table 4). TRAE of grades 1-2 predominated, with an incidence of ≥3-grade TRAE of 5.6% (5/89). These included 1 case of pulmonary infection and 4 cases of myelosuppression. Among all TRAE, myelosuppression was the most common, including leukopenia at 21.3% (19/89), neutropenia at 19.1% (17/89), anemia at 15.7% (14/89), and thrombocytopenia at 7.8% (7/89). Next were alopecia at 20.2% (18/89), pulmonary infection at 14.6% (13/89), and hepatic dysfunction at 10.1% (9/89). The incidence rates of proteinuria, peripheral neurotoxicity, gastrointestinal reactions, and rash were all less than 5%.

**Table 4.** Treatment-related adverse events (TRAE) in all patients.

Treatment-related adverse events (TRAE)	Total *N* (%)	Patients with HER2-positive BC *N* (%)	Patients with HER2-low-expression BC *N* (%)	Grade 3 or 4 *N* (%)
Total	56 (62.9)	31 (34.8)	25 (28.0)	5 (5.6)
Leukopenia	19 (21.3)	2 (2.2)	8 (9.0)	4 (4.5)
Neutropenia	17 (19.1)	12 (13.5)	5 (5.6)	4 (4.5)
Anemia	14 (15.7)	7 (7.9)	7 (7.9)	—
Thrombocytopenia	7 (7.9)	5 (2.2)	5 (5.6)	1 (1.1)
Alopecia	18 (20.2)	11 (12.4)	7 (7.9)	—
Hepatic dysfunction	9 (10.1)	5 (5.6)	4 (4.5)	—
Pulmonary infection	13 (14.6)	8 (9.0)	5 (5.6)	1 (1.1)
Gastrointestinal reactions	3 (3.4)	1 (1.1)	2 (2.2)	—
Interstitial pneumonia	2 (2.2)	1 (1.1)	1 (1.1)	—

Some patients may experience multiple TRAE concurrently.

We also compared the incidence of TRAEs between patients with HER2-positive and HER2-low BC. The occurrence rate of TRAEs was 64.6% (31/48) in patients with HER2-positive BC and 61.0% (25/41) in patients with HER2-low-expression BC, showing no significant difference. Therefore, we further analyzed the incidence of TRAEs among all patients receiving different combination treatment regimens. The TRAE rates in patients receiving monotherapy and those undergoing combination chemotherapy were 54.5% (18/33) and 50% (6/12), respectively, lower than in patients receiving combination-targeted therapy (72.7%, 24/33) and other combination therapies (72.7%, 8/11). Moreover, we compared the TRAE incidence between patients on a 14-day and a 21-day treatment cycle. The results showed that the incidence of TRAEs was 55.9% (38/68) in patients on a 14-day cycle, while it was 85.7% (18/21) in patients on a 21-day cycle.

## Discussion

The objective of this study was to assess the efficacy and safety profile of RC48 in patients with BC. Our findings revealed the following key insights: (1) RC48 demonstrated favorable efficacy and manageable safety in patients with advanced or metastatic BC, particularly showing superior efficacy in patients with HER2-positive BC compared to those with HER2-low-expression. (2) Among patients with HER2-positive BC, those receiving combination-targeted therapies exhibited superior therapeutic responses. Among patients with HER2-low-expression BC, HR-positive individuals demonstrated significantly longer PFS compared with their HR-negative counterparts. (3) Sequential administration of ADC therapy, notably T-DM1 followed by RC48, correlated with extended survival durations compared with the general patient cohort. (4) Considering patient performance status and drug tolerance, some patients had their treatment cycles extended to 21 days, without a statistically significant difference in PFS compared with the standard 14-day regimen. In summary, RC48 manifests promising clinical benefits in BC management, underscoring the necessity for further validation through expansive and protracted investigations.

Our study elucidates that the expression level of HER2 serves as a pivotal determinant of RC48 efficacy. Notably, patients with HER2-positive BC exhibited prolonged PFS (6.6 months vs 4.1 months) and higher ORR (31.3% vs 19.5%) compared with their HER2-low-expression counterparts, underscoring the superior efficacy of RC48 in patients with HER2-positive BC. These findings align with those of previous studies, wherein patients treated with 2 mg/kg RC48 in the C001&C003 pooled analysis achieved a median PFS of 6 months, while those in the HER2-low-expression subgroup attained a median PFS of 5.7 months.^31,32^ Furthermore, noteworthy responsiveness was observed in patients with HER2-low-expression BC in our study, exemplified by a sixth-line patient treated with RC48 in combination with anti-angiogenic therapy, achieving CR and a remarkable PFS of 15.1 months. Therefore, ADC therapy targeting tumors with HER2 low expression has great potential. The results from the DESTINY-Breast06 (DB-06) study further confirm that T-DXd significantly improves PFS in patients with HR+, HER2-low/ultralow advanced BC who have been previously treated with endocrine therapy.^33^ Similar therapeutic efficacy has been noted in patients with HER2-low-expression metastatic BC treated with other ADCs such as SYD985 and SG.^34-36^ Therefore, ADC therapy stands as a promising significant therapeutic modality for advanced HER2-low-expression BC.

Due to the limited sample size of patients who have received prior treatment with other ADC agents, we were unable to conduct precise statistical comparisons. However, among the small subset of patients who received sequential therapy with T-DM1 followed by RC48, their PFS was approximately twice that of the overall population (10.2 months vs 5.5 months). The potential for improved efficacy with sequential use of different ADC agents may be attributed to several factors. First, the diversity in antibody and biological activity of the payload could overcome tumor heterogeneity, thereby expanding the scope of indications.^37^ Second, RC48 may overcome resistance mechanisms to elicit tumor responses due to bystander effects caused by cleavable linkers.^38,39^ Additionally, in another study investigating the sequential use of different ADC agents, the choice of regimen sequence may also influence the final treatment response and prognosis.^40^ As the clinical application of ADC agents continues to expand, further clinical research is warranted to explore the mechanisms of cross-resistance between ADC agents and their implications for clinical guidance.

Furthermore, given the variances in tumor microenvironments across different organs, we specifically investigated the impact of metastatic sites on the treatment response to RC48. However, in our study cohort, no statistically significant differences were observed, possibly due to the presence of concurrent multiorgan metastases in patients. Brain metastases pose a therapeutic challenge in tumor drug delivery due to the blood-brain barrier. Our study revealed that patients with brain metastases had a longer OS than those without brain metastases (6.1 months vs 5.3 months), although statistical significance was not reached. The composition of RC48 includes disitamab conjugated with MMAE via a cleavable linker. The cleaved MMAE may possess permeability across the blood-brain barrier. In previous animal experiments, T-DM1 could penetrate the blood-brain barrier and enter the cranium.^41,42^ The subgroup analysis results of the DB-04 study showed that among 24 patients with HER2-low-expression BC with baseline brain metastases, those treated with T-DXd had an intracranial PFS of 9.7 months and an intracranial ORR of 25%, suggesting that T-DXd may offer a new treatment option for patients with HER2-low BC with brain metastases.^43^ In the future, we will enroll more patients with brain metastases for extracranial and intracranial prognostic evaluation, and further explore the ability of RC48 to improve the prognosis of patients with brain metastases.

In our study, the most common adverse events included myelosuppression and alopecia. Although these adverse events are relatively common, they are generally manageable and controllable. Patients responded well after receiving G-CSF and other supportive agents. The incidence of TRAEs is consistent with previous RC48 clinical trials, demonstrating a relatively stable safety profile.^27^ The toxicity profile of ADCs appears to be associated with the stability of the conjugate in the bloodstream and off-target effects of the payload. In previous clinical studies of RC48, cardiac toxicity was not observed through monitoring of cardiac enzyme profiles, BNP levels, and echocardiography. Therefore, according to the review of medical records, patients receiving RC48 did not routinely undergo cardiac monitoring. In similar ADC drugs, ILD has been associated with treatment with T-DXd, necessitating close monitoring of respiratory signs and symptoms in patients.^44,45^ In our study, only 2 cases of ILD (2.2%) were observed. The potential mechanism of lung injury is related to the independent uptake of T-DXd by alveolar macrophages,^46^ while further investigation is needed to elucidate the mechanism of RC48.

This study is subjected to several limitations that warrant consideration. First, its retrospective design precludes control over exposure factors, thereby allowing only for correlations rather than causal inferences. Second, real-world studies are susceptible to more uncontrollable confounders, potentially influencing outcomes. Limited usage of the medication in real-world scenarios has resulted in a smaller sample size, possibly introducing bias. It is challenging to evaluate and quantify patients’ quality of life using standardized scales. Additionally, the study primarily focuses on RC48 monotherapy efficacy without establishing comparative drug control groups, making direct regimen comparisons unfeasible. Regarding survival time analysis, we used the RMST method; however, due to incomplete observation period maturity, this may affect survival time accuracy. To comprehensively assess RC48 efficacy and safety, ongoing patient follow-up and increased sample size are essential for further analysis.

## Conclusion

In summary, our study confirms that RC48 demonstrates favorable efficacy and manageable toxicity in patients with both HER2-positive and HER2-low-expression BC. Moreover, in HER2-positive BC, combination therapy with targeted drugs showed superior efficacy, while in patients with HER2-low-expression BC, the HR status may influence the final outcomes. Moreover, as a follow-up treatment option post other ADC therapies, RC48 demonstrates encouraging efficacy. Notably, in our study, RC48 was predominantly used in later-line treatments. We eagerly anticipate additional clinical research exploring the utilization of RC48 in first- and second-line therapies for patients with low HER2 expression, with the goal of assessing its clinical effectiveness.

## Supplementary material

Supplementary material is available at *The Oncologist* online.

oyae304_suppl_Supplementary_Figure_S1

## Data Availability

The datasets are available from the corresponding author upon reasonable request.
