# Supplementary material for: Real-world application of disitamab vedotin (RC48-ADC) in patients with breast cancer with different HER2 expression levels: efficacy and safety analysis
Source: Oncologist. 2024 Nov 16;30(8):oyae304. doi: 10.1093/oncolo/oyae304 (PMC12395257; doi:10.1093/oncolo/oyae304)
Supplement: oyae304_suppl_Supplementary_Figure_S1 [file oyae304_suppl_supplementary_figure_s1.docx]

**
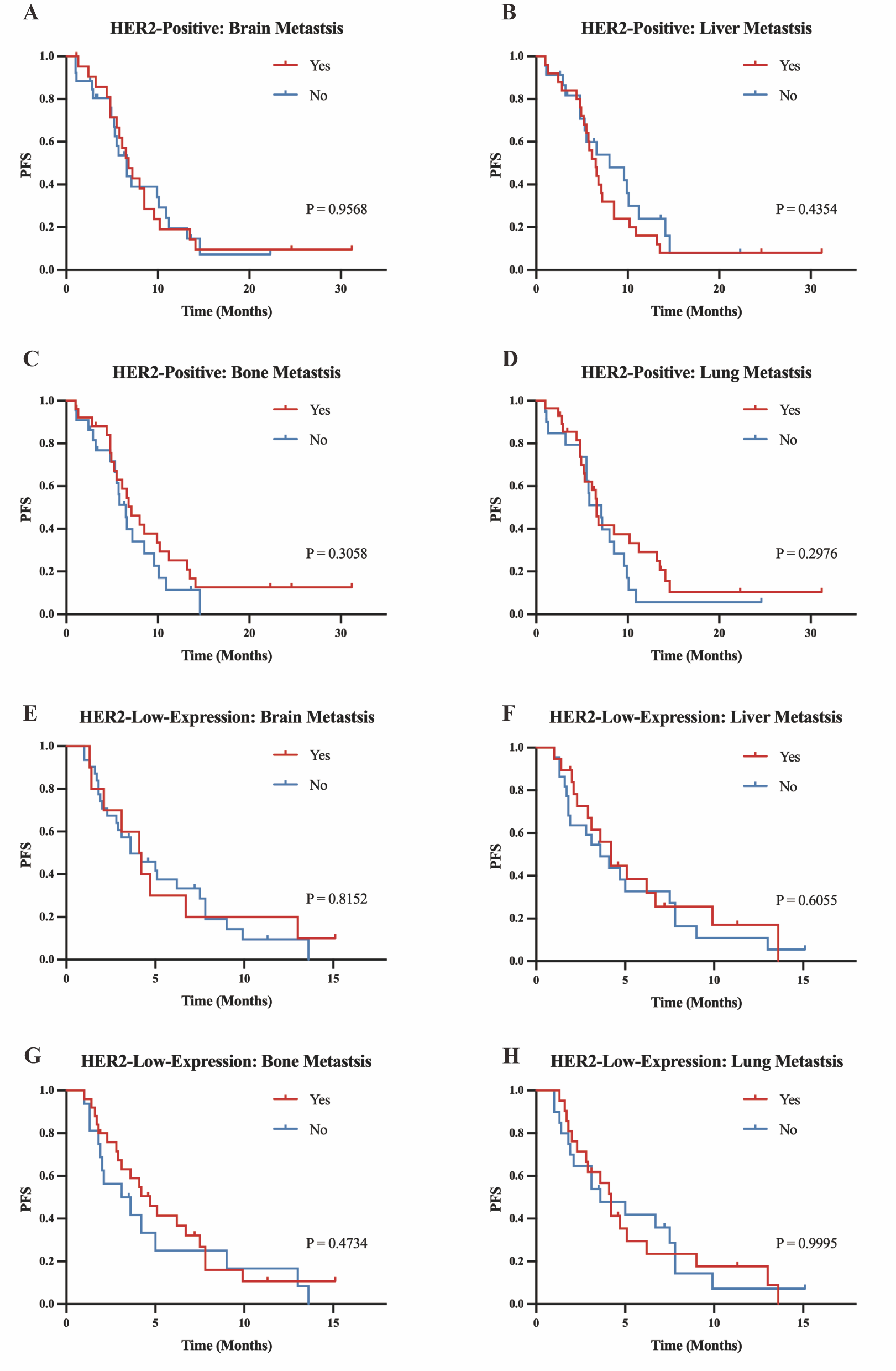
Supplemental Figure S1. Survival plots of subgroups with different HER2 expression levels.** Comparison of PFS in HER2-positive breast cancer patients with or without brain metastasis(A), liver metastasis(B), bone metastasis(C), lung metastasis(D). Comparison of PFS in HER2-low-expression breast cancer patients with or without brain metastasis(E), liver metastasis(F), bone metastasis(G), lung metastasis(H).
